# Targeted delivery of lysosomal enzymes to the endocytic compartment in human cells using engineered extracellular vesicles

**DOI:** 10.1038/s41598-019-53844-5

**Published:** 2019-11-21

**Authors:** Mai Anh Do, Daniel Levy, Annie Brown, Gerard Marriott, Biao Lu

**Affiliations:** 1 Department of Bioengineering, School of Engineering, Santa Clara University, 500 El Camino Real, Santa Clara, California 95053 USA; 2 Department of Bioengineering, University of California at Berkeley, Berkeley, CA94720 USA

**Keywords:** Molecular medicine, Nanoparticles

## Abstract

Targeted delivery of lysosomal enzymes to the endocytic compartment of human cells represents a transformative technology for treating a large family of lysosomal storage diseases (LSDs). Gaucher disease is one of the most common types of LSDs caused by mutations to the lysosomal β-glucocerebrosidase (GBA). Here, we describe a genetic strategy to produce engineered exosomes loaded with GBA in two different spatial configurations for targeted delivery to the endocytic compartment of recipient cells. By fusing human GBA to an exosome-anchoring protein: vesicular stomatitis virus glycoprotein (VSVG), we demonstrate that the chimeric proteins were successfully integrated into exosomes which were secreted as extracellular vesicles (EVs) by producer cells. Isolation and molecular characterization of EVs confirmed that the fusion proteins were loaded onto exosomes without altering their surface markers, particle size or distribution. Further, enzyme-loaded exosomes/EVs added to cultured medium were taken up by recipient cells. Further, the endocytosed exosomes/EVs targeted to endocytic compartments exhibited a significant increase in GBA activity. Together, we have developed a novel method for targeting and delivery of lysosomal enzymes to their natural location: the endocytic compartment of recipient cells. Since exosomes/EVs have an intrinsic ability to cross the blood-brain-barrier, our technology may provide a new approach to treat severe types of LSDs, including Gaucher disease with neurological complications.

## Introduction

Lysosomal storage diseases (LSDs) are a group of over 50 inherited metabolic disorders that have a devastating effect and impose a heavy burden on both the patient and the health care system^1–3^. Although many of these disorders are rare, the combined incidence is high with an estimation of 1 in 7,000~8,000 live births^4^. LSDs are heterogeneous; each one is caused by a defect to a different lysosomal enzyme. The loss of these enzymatic activities leads to accumulation of undegraded substrates, which can be toxic to cells and result in multiple organ damage^5^. The clinical signs and symptoms include facial dimorphism, hepatosplenomegaly, ocular impairment, skeletal and cardiovascular involvement, as well as hematological abnormalities. Moreover, two-thirds of patients with LSDs manifest distinct neurological symptoms, ranging from progressive neurodegeneration to severe cognitive impairment as well as epilepsies, behavioral abnormalities and/or psychiatric disorders^6^.

Current options for treating hereditary LSDs are limited. They include specific enzyme replacement therapy (ERT) with recombinant human enzymes or a handful of supportive measures^7–9^. ERT was first used to replace defective enzymes for Gaucher’s patients in the 1990s^10,11^. Subsequently, this approach became the standard of care and has extended to other LSDs including Fabry disease, Pompe disease, mucopolysaccharidosis, metachromatic leukodystrophy, and acid lipase deficiency^12–16^. Although ERT is effective in reversing the visceral and hematologic manifestations associated with the mild form of the disease, critical limitations have emerged, including insufficient reversal of the pathology in tissues such as muscle, bone, cartilage, heart and lung^17^. Importantly, ERT has little effect on cases with severe neurological complications, putatively due to difficulty crossing the blood-brain barrier^8,18^. Although there are some promising outcomes recently to increase enzyme delivery to the brain, these methods tend to be more invasive and require additional improvements^19,20^. One solution to the current drawbacks of protein therapy to Gaucher treatment is to develop delivery systems that can both reach and penetrate barriered tissues, such as the brain. Ideally, these systems would deliver functional enzymes to their native action sites, namely the endocytic compartments of diseased cells.

EVs are cell-derived nano-vesicles that play an important role in mediating cell-to-cell communication^21–23^. These vesicles have been shown to shuttle a large number of macromolecules, including proteins, enzymes, nucleic acid and lipids to various tissues and organs^24,25^. Due to their intrinsic tissue-penetrating ability, EVs represent a more desirable delivery tool over other existing technologies such as liposomes, synthetic polymers and gold nanoparticles^26,27^. In fact, engineered EVs, particularly exosomes (the subpopulation of EVs derived from multiple vesicular bodies), have been shown to deliver RNA therapeutics or other drugs to both brain tissues and deep-seated cancer cells^28–31^. Recently, we have developed a decoy exosome system that functions as a biological sponge to antagonize tumor-necrosis factor alpha^32^. In this previous study, we tested the hypothesis that engineered EVs or exosomes and related biologics can function as an effective extracellular sponge binding to inflammatory agents in the extracellular space. In exploiting EVs for targeted delivery of bioactive enzymes to an intracellular site for the treatment of human diseases^33^, one abstract reported the preliminary work on the potential use of opto-genetically engineered exosomes and β-glucocerebrosidase (GBA) for the treatment of Gaucher disease^34^

Human β-glucocerebrosidase (GBA) is an important well-characterized lysosomal enzyme that localizes within the endocytic compartment of human cells. Defective forms of GBA are responsible for Gaucher disease and previous studies have shown that the introduction of functional GBA can effectively reverse disease symptoms^35,36^.

Here, we describe a *de novo* method to load GBA onto exosomes by developing a genetic fusion protein using an exosome targeting transmembrane protein, VSVG. These GBA-loaded exosomes can be isolated in a pure form from conditioned medium. The isolated exosomes/EVs are added to the medium of targeted recipient cells, where they effectively deliver bioactive GBA to their endocytic compartments.

## Methods

### Materials

Reagents were obtained from the following commercial sources: recombinant human glucosylceramidase/GBA protein (R&D Systems/Bio-Techne; Minneapolis MN); 4-methylumbelliferyl-beta-D-glucopyranoside, sodium cholate, glycine, citric acid, DTT, and NaOH (Sigma Aldrich; St. Louis, MO); Human embryonic kidney cell line HEK293 (Alstem; Richmond, CA); Human glioblastoma cell line U87 (ATCC; Manassas, VA); Human hepatocellular carcinoma cells, HepG2 (ATCC; Manassas, VA); Lipofectamine2000 (Thermo Fisher Scientific; Waltham, MA); FuGENE6 (Promega; Madison, WI); Dulbecco’s Modified Eagle Medium (DMEM), fetal bovine serum (FBS), UltraCULTURE (Lonza; Allgendale, NJ); LysoTracker Red DND-99, CellLight Early Endosomes-RFP/Late Endosome-RFP BacMam 2.0, polyclonal TurboGFP primary antibody, goat anti-rabbit horseradish peroxidase conjugated secondary antibody (Invitrogen; Carlsbad, CA); Dot-blot antibody arrays, ExoQuick-TC exosome precipitation solution (SBI; Palo Alto, CA); Prestained protein markers and precast 4~12% SDS-PAGE (GenScript; Piscataway, NJ); Hoechst 33342 and Pierce ECL Western Blotting Substrate (ThermoFisher Scientific; Fremont, CA).

### Vector design and construction

The mammalian vector expressing the VSVG-GFP fusion gene was constructed as previously described [28]. The full-length coding sequence of human GBA (XM_006711270) was purchased from Genscript (Piscataway, NJ). The partial GBA coding sequence was inserted into two locations of the VSVG-GFP vector, yielding two construct designs, GBA-VSVG-GFP and VSVG-GFP-GBA, depending on whether GBA were appended at the N- or C- terminus of the VSVG-GFP vector respectively. The final constructs were verified by double-stranded DNA sequencing (GenScript; Piscataway, NJ); their encoded chimeric proteins were annotated and provided (Supplementary Sequences).

### Cell culture and transfection

Human cells (HEK293, U87 and HepG2) were cultured in high glucose Dulbecco’s Modified Eagle Medium (DMEM) supplemented with 10% FBS, 2 mM glutamine and 100 U/mL penicillin/streptomycin (Gibco; Manassas, VA). Cells were maintained at 37 °C and 5% CO_2_. Cells were transiently transfected using either Lipofectamine 2000 transfection reagents. Typically, 1.5~2 μg of plasmid DNA was used to transfect cells (40~60% confluency) in each well of a 6-well plate. After 24~48 hours, over 80% of cells were effectively transfected.

### Nuclear and lysosomal staining

Cultured cells on glass bottom dishes were incubated with a PBS-diluted Hoechst 33352 stain (1:1000 dilution) for 10 minutes at 37 °C. The Hoechst solution was removed and cells were washed with PBS before imaging via confocal microscopy to show nuclear staining (blue). Similarly, cells were stained with a dilution of 75 nM LysoTracker Red DNA-99 in cultured medium and incubated at 37 °C for 30 minutes. The LysoTracker solution was then replaced with either fresh culture medium or PBS before imaging via confocal microscopy to show lysosomal staining (red).

### Extracellular vesicle preparation

A combination of centrifugation, ultrafiltration and precipitation was used to prepare EVs/exosomes as described^37^. Briefly, HEK293 cells cultured in 10% FBS supplemented complete medium were switched to serum-free UltraCULTURE for 48 hours. The conditioned medium was then collected, centrifuged at 1500xg for 10 min and filtered through a 0.2 µm syringe filter to remove cell debris and large extracellular vesicles ( > 200 nm in diameter). Finally, EV-containing medium was mixed with the ExoQuick-TC solution (1:4 dilutions) and incubated overnight at 4 °C. The precipitate was then collected by centrifugation at 3,000xg for 90 min at 4 °C. The EV pellet was resuspended in PBS and stored at −20 °C.

### Nanoparticle tracking analysis (NTA)

EVs isolated from modified producer cells were subjected to nanoparticle tracking analysis (NTA) using a NanoSight LM10 instrument (Malvern Instruments Ltd; Malven, UK) with a 405 nm and 60 mV laser source as previously described [31]. Typically, 1 ml of a diluted exosome preparation was used for the laser light scattering study. The instrument rendered 3 recordings per sample at 60 seconds each; NTA software was then used to determine the size distribution of exosomes.

### Western blot and dot-blot analysis

Whole cell lysates (WCLs) were prepared from either control or transiently transfected HEK293 cells^38^. 30–150 μg of proteins from either exosome or WCLs were loaded on a precast gradient gel (4~12%) and blotted on a polyvinylidenedifluoride membrane. The membrane was blocked for 1 hour at room temperature before overnight incubation at 4 °C with anti-rabbit Turbo-GFP antibody (1:2000) and subsequently with horseradish peroxidase conjugated goat anti-rabbit secondary antibody (1:4000) for 1 hour at room temperature. The membrane was visualized with Pierce ECL Western Blotting Substrate on ImageQuant LAS 500 imager (GE Healthcare Life Sciences; Issaquah, WA).

A dot-blot kit from SBI (Palo Alto, CA) was used to identify target proteins as described^39,40^. Each blot contained 12 pre-printed spots and featured 8 antibodies against exosomal markers including CD63, CD81, ALIX, FLOT1, ICAM1, EpCam, ANXAS and TSG101, as well as a negative control, cytosolic GM130 to identify any cellular contamination. 100~200 μg of exosomal proteins were used in each assay according to the immuno-binding and detection protocol described in the user manual.

### Enzyme assays

GBA activity was determined fluorometrically using 4-methy-lumbelliferyl-beta-D-glucopyranodide (NBD-Glu) as a substrate^40,41^. For each assay, the NBD-Glu solution was freshly prepared and positive controls (recombinant human GBA) was included. Typically, the enzymatic reaction was carried out in a volume of 50 µl, using either 150 µg (WCLs) or 50 µg (exosomes), in a pH 6 solution containing 50 mM sodium citrate, 25 mM sodium cholate and 5 mM DTT. The reaction was incubated for 30 min at 37 °C and stopped by adding 50 µl of stop solution (0.5 M glycine, 0.3 NaOH, pH 10). The fluorescent signal at a wavelength of 365 nm excitation and 445 nm emission was recorded using a TECAN infinite M200PRO plate reader.

### Fluorescence-activated cell sorting (FACS) analysis

Equal amounts of exosomes were chemically stained using a green fluorescent dye, Exo-Glow (SBI, Palo Alto, CA) according to the manufacturer’s instructions. 0.3 µg/μl of stained exosomes were added into cultured media of HEK293 cells to allow for cellular uptake during 48 hour incubation periods. The cells were then washed, trypsinized and re-washed using PBS. The corresponding cells were then subjected to FACS analysis using channels to detect GFP fluorescence^42^.

### Fluorescence and confocal microscopy

For live cell experiments, cultured cells were imaged using an Olympus fluorescence microscope (Waltham, MA), or a Leica TCS SP8 confocal microscope (Buffalo Grove, IL). Images were taken at indicated time-points using the same exposure conditions within the group for comparison. To show the intracellular localization of the fluorescent fusion proteins, both fluorescence and phase/TLD images were overlaid using the Leica Las X imaging software.

### Data collection, analysis and statistics

The student’s t-test was used to determine statistical significance of our studies, with P values < 0.05 being considered significant. All values were expressed as mean ± standard deviation.

## Results

### System design and exosome loading strategy of lysosomal enzyme GBA

**Figure 1:**
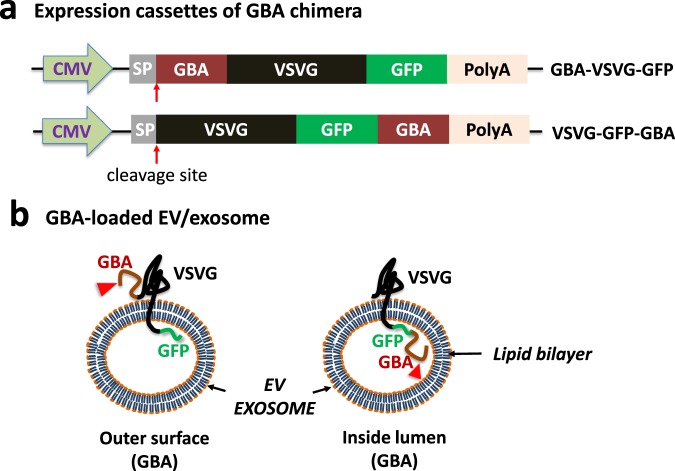
Design of GBA fusion proteins for exosome targeting and anchoring. (**a**) Schematic illustration of expression cassettes for two GBA fusion genes. CMV: cytomegalovirus promoter; SP: signal peptide coding sequences, where the red arrows indicate the cleavage site of the SP; PolyA: polyadenylation signaling. (**b**) GBA-loaded exosome illustration. The red arrow heads indicate different localization of GBA enzymes on exosomes according to the topology of VSVG on the lipid bilayer. The mature GBA is situated on the outer surface of exosome when GBA is fused at the N-terminus of VSVG, while GBA is situated within the lumen of the exosome when tagged at the C-terminus of VSVG.

We have previously developed a method that enables protein loading onto exosomal targeting and anchoring protein, VSVG^42^. Using a genetic fusion approach, it is possible to utilize intracellular targeting and anchoring of a protein payload to the exosomal compartments of human cells. In this study, this approach was used to load the lysosomal enzyme GBA onto exosomes by fusing GBA with VSVG-GFP (Fig. 1a). The coding sequence of mature human GBA was inserted in one of two locations, creating two distinct fusion genes, whose expression was controlled by a CMV promoter (Fig. 1a). To ensure fusion proteins were anchored to the exosomal membrane with the correct spatial topography, we left the signal peptide (SP) sequence and the transmembrane helix of VSVG fully intact in all constructs. The first fusion gene (Upper, Fig. 1a) was expected to produce a protein chimera with GBA at the N-terminus of the VSVG-GFP, while the second (Lower, Fig. 1a) should create a chimera with GBA at the C-terminus after the GFP. When incorporated in exosomes, the first chimera should display the GBA enzyme on the outer surface of exosome, while in the second chimera, GBA would localize within the lumen of the exosomes (Fig. 1b). We fused a fluorescent GFP to all constructs, which allowed us to observe and quantify molecular trafficking of the gene product in human cell cultures. Compared to earlier studies on exosome-engineering, we can identify several innovations: the loading of functional GBA enzymes with defined geometry on the exosome surface and the integration of a sensitive probe for high-resolution imaging of distributions of the engineered exosomes in live cells^28,42,43^.

### Loading bioactive GBA onto exosomes in living human cells

**Figure 2:**
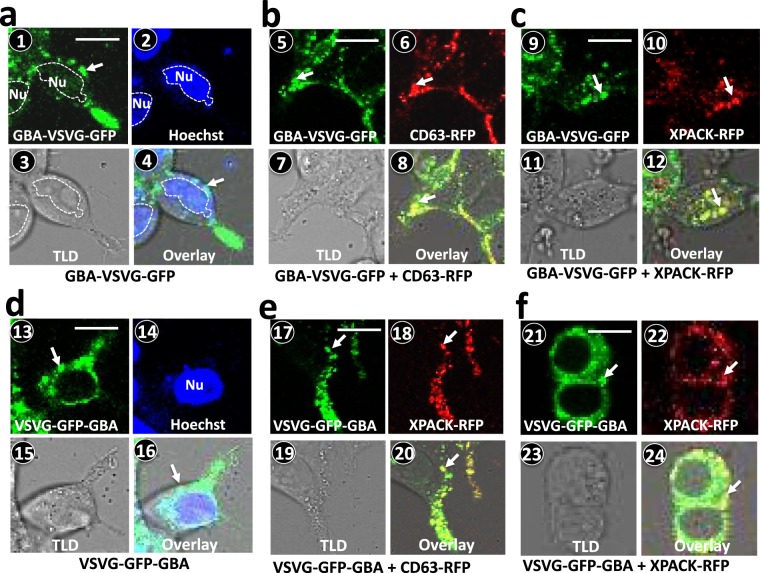
Molecular trafficking of GBA-fusion proteins in genetically modified HEK293 cells. HEK293 cells were transfected with either GBA-VSVG-GFP alone (**a**) or co-transfected with either endosomal marker CD63-RFP (**b**) or XPACK-RFP (**c**) for 72 hours. Confocal images of live cells expressing GBA-VSVG-GFP (**a1**; green, arrow) and with Hoechst nuclear stain (**a2**, blue) and their overlay (**a4**) are shown. GBA-VSVG-GFP (green; **b5**, **c9**) is co-expressed with either CD63-RFP (**b6**) or XPACK-RFP (**c10**) as shown by their co-localization in overlay images (**b8**, **c12**). Parallel experiments were performed using VSVG-GFP-GBA, and similar results were obtained showing the expression of VSVG-GFP-GBA (**d13–16**) and co-expression with either CD63-RFP (**e17–20**) or XPACK-RFP (**f21–24**). Scale bar represents 10 µm. Arrows indicate subcellular locations of fusion protein. Nu: nucleus.

First, we investigated the subcellular localization of GBA fused to the VSVG scaffold and imaged the GFP to visualize its intracellular trafficking to exosomes in live HEK293 cells. The cells were transfected with genetic constructs as detailed in the Methods section and imaged via confocal fluorescence microscopy for up to 3 days to assess GFP expression patterns and intracellular localization. Analysis of these images showed that within 24-hours of post-transfection with the GBA-VSVG-GFP construct, GFP fusion proteins (green, arrow) localized to puncta (Fig. 2,a1). The cytosolic localization of these intracellular fluorescent proteins was evident in an overlay of fluorescence and phase contrast images (Fig. 2,a4). This finding was consistent with the localization of the fusion protein to endocytic structures. Similar results were obtained when cells were transfected with VSVG-GFP-GBA (Fig. 2,a13–16). Together these data indicated that the protein chimeras including the GBA appendages were efficiently expressed and properly directed to exosomes in transfected cells.

We next investigated whether these displayed fluorescent VSVG fusion proteins were correctly integrated into pre-exosomal compartments during biogenesis. For this purpose, HEK293 cells were co-transfected with one of the GBA-VSVG-GFP constructs and one of two validated exosomal markers, namely CD63-RFP or XPACK-RFP. Confocal images of transfected cells were recorded for 72 hours after co-transfection. The results revealed extensive overlap of green and red fluorescence signals of GBA-VSVG-GFP co-transfected with either CD63-RFP (Fig. 2b5–8) or XPACK-RFP (Fig. 2c9–12**)**. We obtained similar results in cells expressing VSVG-GFP-GBA and either CD63-RFP (Fig. 2e17–20) or XPACK-RFP **(**Fig. 2f21–24**)**. Together, our data strongly indicate that both GBA protein chimeras successfully targeted and incorporated themselves onto exosomes before their release to extracellular environments.

**Figure 3:**
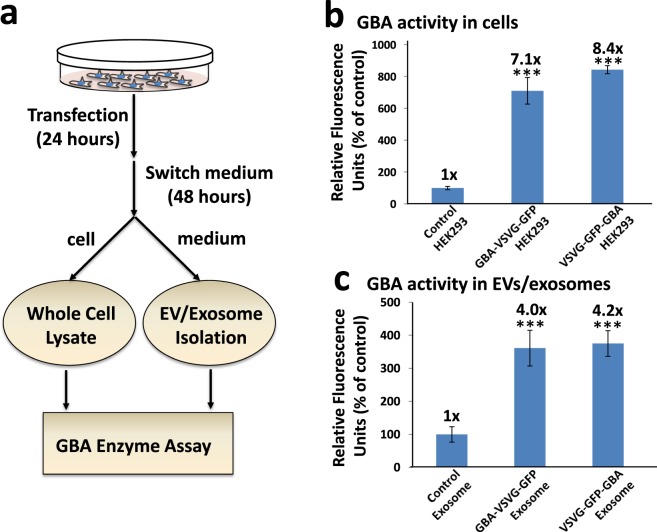
Enzyme assay protocol and GBA activities in whole cell lysates and exosomes. (**a**)Workflow of preparation of whole cell extracts and exosomes for a GBA enzyme assay following transfection of HEK293 cells with either GBA-VSVG-GFP, VSVG-GFP-GBA, or control (unmodified plasmid) for 24 hours. At the end of transfection, cells were collected and both the whole cell lysates and exosomes were prepared for a GBA enzyme assay. The relative GBA activity in whole cell extracts (**b**) and in exosomes (**c**) is shown. All values are expressed as mean ± standard deviation (n = 3). ***p < *0*.*001 significant vs control*, using a student-t test.

Post-translational modification of GBA such as glycosylation is an important step in the production of a functional enzyme^44,45^.To assess whether our expressed enzymes were active post-exosomal loading, transfected cells were cultured in serum free media and modified exosome were isolated as described. GBA activity was then measured from isolated exosomes. Whole cell lysates (WCL) were prepared from the transfected cells as detailed in the Methods section. As shown in Fig. 3b, the relative GBA activities in the WCL drastically increased by 7.1- and 8.4-fold for GBA-VSVG-GFP and VSVG-GFP-GBA respectively, compared to a mock control (P < 0.001). As expected, the GBA activities in isolated exosomes also increased by 4.0- and 4.2-fold compared to those mock controls for GBA-VSVG-GFP and VSVG-GFP-GBA respectively (Fig. 3c, P < 0.001).

In summary, our data support that VSVG serves as a molecular scaffold that can both target and load their appended GBA and GFP components to exosomes in HEK293 cells. The readily detectable GFP signal in transfected cells suggests that the loading of both GBA and GFP is robust. Moreover, the drastically increased levels of GBA activity demonstrate that the enzyme payloads in both transfected producer cells and modified exosomes remain active.

### Characterization of engineered EVs

**Figure 4:**
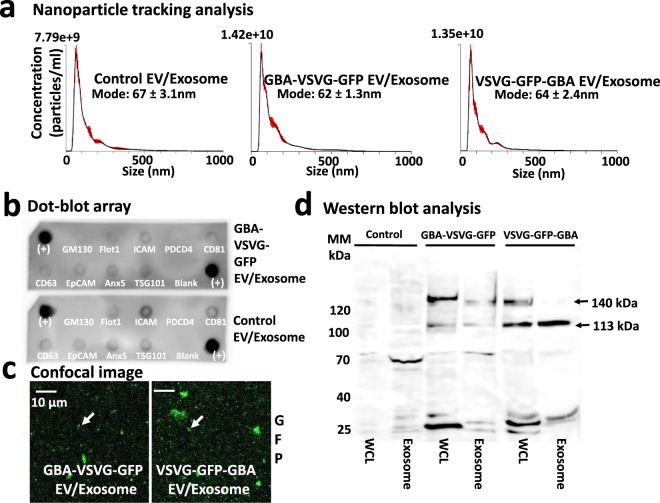
Characterization of engineered exosomes. HEK293 cells were transfected with either GBA-VSVG-GFP, or VSVG-GFP-GBA, or mock control (control plasmid) for 72 hours, and exosomes were prepared and subjected to nanoparticle-tracking analysis (**a**), a dot-blot array analysis (**b**), confocal images (**c**) and Western blot analysis. (**d**) As shown in (**a**), there is no difference of particle size and distribution between these exosomes from control cells a GBA-VSVG-GFP or VSVG-GFP-GBA transfected cells. Dot-blot array analysis of 9 exosomal markers also showed similar expression pattern of engineered exosomes from cells transfected with GBA-VSVG-GFP (**b**; upper panel) vs. non-modified control (**b**; lower panel). (**c**) Confocal images of GBA-VSVG-GFP and VSVG-GFP-GBA modified exosomes. Western blot analysis showed the expression of GBA-VSVG-GFP and VSVG-GFP-GBA fusion proteins in transfected cell lysates or exosomes, compared with background levels of that in a mock control. (**d**) Arrows indicates the predicted size of the trimeric (GBA + VSVG + GFP) fusions (~140 kDa), and possibly differently post-translational modification or partially de-gradated bands (~113 kDa).

We next characterized isolated EVs to assess whether the gene-product modifications affected their physical or biochemical properties. HEK293 cells were cultured and transfected with either construct for 72 hours, before WCL and EVs preparation as described earlier. First, we verified the size and particle distribution of the modified EVs were consistent with those of native (control) EVs, using a NanoSight NS80 system. As shown in Fig. 4, the peak size of 62–64 nm of exosomes modified by either GBA-VSVG-GFP (middle panel) or VSVG-GFP-GBA (right panel) did not differ from that measured in native (control) EVs (left panel).

To further confirm that the purified product consisted of exosomes rather than other EVs, we performed an immune dot-blot analysis on isolated exosomes using a panel of 9 anti-exosomal antibodies following the manufacturer’s protocol. The blot revealed positive stains for 7 out of 8 exosome markers including FLOT1, ICAM1, CD81, CD63, EpCam, ANX5, and TSG101 for GBA-VSVG-GFP modified exosomes (Fig. 4b, Upper Panel) as well as native (control) (Fig. 4b, Lower Panel). The control cytosolic marker (GM130) was negative on both dot-blots, an indication of high purity of our exosome preparations. The same dot-blot but with different exposure times (Fig. S1) can be found in the Supplementary Information.

Next, we investigated whether our isolated EVs, exosomes, were properly loaded with the genetically encoded chimeric proteins. As shown in Fig. 4c, confocal fluorescence images of purified exosomes presented strong GFP expression for both GBA-VSVG-GFP and VSVG-GFP-GBA modified exosomes, indicating that the chimeras were appropriately targeted and anchored to exosomes. To further demonstrate that the full-length chimeras were properly expressed in transfected cells and integrated into secreted exosomes, we performed Western-blot analysis on both WCL and modified exosomes. Using an anti-TurboGFP antibody, we identified a ~140 kDa band was apparent in both GBA-VSVG-GFP and VSVG-GFP-GBA transfected cells but not in mock control samples (Fig. 4d**)**, suggesting specific expression of the full-length chimeras in isolated exosomes and transfected producer cells. An uncropped immune-blot (Fig. S2) can be found in the Supplementary Information. Additionally, we notice some smaller positive bands (such as ~113 kDa) in both WCL and exosome samples, which may have resulted from differential post-translational modifications or partial degradations during sample preparation or exosome loading.

Together, these data demonstrate that our chimeras were properly expressed and integrated into EVs, most likely exosomes. These exosomes were then were released into conditioned medium and isolated with other EVs, after which they still retained their activity.

### Targeted delivery of GBA to the endocytic compartment of recipient cells

**Figure 5:**
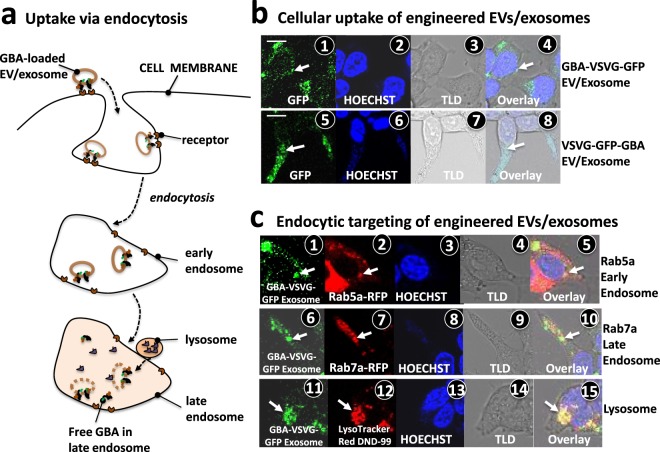
Delivery of GBA enzyme into endocytic compartments of recipient cells. (**a**) Schematic illustration of cellular uptake and lysosomal release of engineered GBA via the endocytosis process. (**b**) Parental HEK293 cells were treated with either GBA-VSVG-GFP (**b1–4**) or VSVG-GFP-GBA (**b5–8**) loaded exosomes in a glass bottom culture dish. 48 hours post-treatment, fluorescent, Hoechst stained, and PMT-Trans images were taken and overlaid (**b1** and **b8**). In a set of separate experiments, GBA-VSVG-GFP or VSVG-GFP-GBA loaded exosomes were used to treat HEK293 cells that were either transfected with RFP-labeled early/late endosome reporters, Rab5a-RFP and Rab7a-RFP (**c1–10**) or stained with a red fluorescent-dye specific to lysosomes (**c11–15**). Arrows indicate early/late endosomes, or lysosomal structures. Scale bar 10 μm.

Next, we investigated the intracellular delivery of GBA via engineered exosomes to human recipient cells using a combination of confocal monitoring and enzymatic assays. We first performed confocal imaging on HEK293 cells to track the distributions of these GBA chimeras after internalization. We observed cellular uptake in a concentration and time-dependent manner. The uptake was initially noticeable after 3~6 hours and became attenuated and plateaued after 24 hours (Levy D *et al*. unpublished data). Exosomes were presumed to enter cells via receptor-mediated endocytosis through surface protein interactions (Fig. 5a)^46^. Because VSVG ligand binds strongly to the VLDL-receptor, which appears on the surface of all human cell types, it was expected that VSVG-modified exosomes could effectively enter recipient cells, ultimately targeting their endocytic compartments^46^. In the case of GBA-VSVG-GFP modified exosomes, GBA enzymes would situate on the outer surface of exosomes, thus allowing for degradation of their substrates within the endosomal lumen. We further hypothesized that in the case of VSVG-GFP-GBA, the enclosed GBA enzyme would be released after the integration of modified exosomes into late endosomes (Fig. 5a). To determine the subcellular locations of modified exosomes post-uptake by recipient cells, we first added the either GBA-VSVG-GFP or VSVG-GFP-GBA loaded exosomes into the culture medium of HEK293 cells. We monitored the cellular up-take processes up to 72 hours using fluorescence microscopy. The punctated GFP-fluorescence signal within recipient cells was visible as early as 6 hours after uptake, with increasing intensity for 48 hours and persisted at least72 hours. Confocal images of cellular uptake of modified GBA-VSVG-GFP (Fig. 5,b1–4**)** and VSVG-GFP-GBA (Fig. 5,b5–8) endosomes recorded at 48 hours clearly identified punctated distributions of fluorescent label in the cytosol, which would be consistent with endocytic compartmentalization post-uptake. This cellular uptake was also observed in two additional human cell types, including human glioblastoma cell line U87 (Fig. S3a in Supplementary Information) and human hepatoma cell line HepG2 (Fig. S3b in Supplementary Information). To further verify whether these puncta were truly endocytic components, we carried out a co-localization assay using known markers for either early endosome, late endosomes or lysosomes. We identified endosomes by transfecting HEK293 cells with either early (RAB5a-RFP) or late endosome (RAB7a-RFP) markers before adding GBA-VSVG-GFP loaded exosomes into culture medium for cellular uptake. Confocal images revealed significant co-localization (yellow) of uptake exosomes (green) with red endosomal markers of early endosomes (Fig. 5c1–5) and late endosomes (Fig. 5c6–10). In a separate study, we first added GBA-VSVG-GFP loaded exosomes (green) to HEK293 cells to allow for cellular uptake for 24 hours before staining the cells with LysoTracker Red DND-99 to indicate lysosomal compartments. Analysis of these images showed that the engineered exosomes colocalized with lysosomal marker probe (Fig. 5c11–15). This finding supports our view that the internalized exosomes ultimately migrate to lysosomes. Together, these results demonstrate that our genetically modified exosomes were able to target endocytic compartments after entering recipient cells.

**Figure 6:**
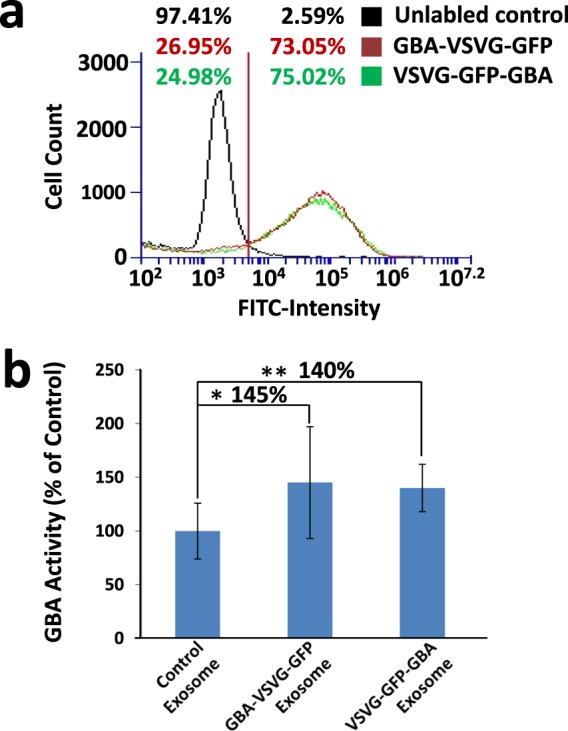
Intracellular delivery of bioactive GBA into living HEK293 cells. (**a**) Parental HEK293 cells were treated with either GBA-VSVG-GFP or VSVG-GFP-GBA modified exosomes for 48 hours before being subjected to FACS analysis. Right shifts in fluorescent signals for both GBA-VSVG-GFP (red line) and VSVG-GFP-GBA (green line) were observed as compared to control cells treated with unlabeled exosomes (black line). (**b**) HEK293 cells were treated with either isolated engineered exosomes or unmodified control exosomes for 48 hours, then washed and lysed. Equal amounts of proteins from the whole cell extracts were subjected to GBA enzymatic assays as described in the Materials and Methods. Values were expressed as mean ± standard deviation from 3 independent experiments (n = 11). *p < *0*.*05*, **p < *0*.*01* significant vs control using a student-t test.

Next, we quantified the effectiveness of engineered exosomes to enter recipient cells by using FACS analysis. Following transfection of the cells with our engineered chimeras, we isolated exosomes expressing either the GBA-VSVG-GFP or VSVG-GFP-GBA and further labeled these preparations with a green fluorescent dye to enhance the sensitivity of FACS detection. We introduced the fluorescent dye-labeled exosomes to cultured HEK293 cells for 48 hours and conducted FACS to evaluate exosome uptake. When compared to unlabeled control exosomes, the uptake rates of both GBA-VSVG-GFP and VSVG-GFP-GBA were similar and equal to the control values of 73~75%. Overall, the uptake into HEK293 cells showed a 50-fold increase in fluorescent intensity over the unlabeled control (Fig. 6a). Thus, FACS data independently confirmed that our GBA-loaded exosomes are effective in gaining entry to recipient cells.

Next, we tested whether GBA remained enzymatically active after internalization in recipient cells. We conducted an enzyme assay to quantify the levels of GBA activity in engineered exosomes post-cellular uptake and compared these results to an unmodified control group. Equal amounts of GBA-VSVG-GFP, VSVG-GFP-GBA and unmodified control exosomes were added to HEK293 cells for 48 hours. Subsequently, GBA activities from the WCL and the resultant activities normalized to the control group (set as 100%). As shown in Fig. 6b, both the GBA-VSVG-GFP and VSVG-GFP-GBA modified exosomes exerted significantly greater GBA activity by ~45% (P < 0.05) and ~40% (P < 0.01) respectively, compared to the unmodified control. Thus, our data indicate that the genetically modified exosomes taken up by recipient cells display functionally active GBA.

In summary, the methods we have described to load GBA on the outer surface and inner lumen of exosomes produces functional enzymes that are properly directed to endocytic compartments within recipient cells. The highly efficient delivery and internalization of our modified exosomes was shown to significantly enhance GBA activity within these cells.

## Discussion

We have developed a genetic method to produce engineered exosomes that deliver functional lysosomal enzymes to the endocytic compartments of recipient cells. Unlike other nanoparticle-based carriers, exosomes are produced in human cells and exhibit intrinsic and specific mechanisms to enter the cytosol of living cells both *in vitro* and *in vivo*. Consequently, engineered exosomes hold promise in delivering protein payloads to targeted cells that are deep-seated in brain tissue^28^. Using this method, we effectively demonstrated that our engineered exosomes successfully delivered GBA, an enzyme associated with LSD, to human cells, where they target endocytic compartments. Our method may provide an alternative and more effective treatment option to manage LSDs, the largest family of human metabolic diseases. That includes Gaucher disease, which has been linked to neurological complications.

Over the past decade, there have been significant advances in engineering exosomes as drug delivery vehicles. The ability to use exosomes to deliver therapeutic cargo, including siRNA, mRNA, proteins, and small molecule drugs presents new opportunities for targeted delivery to treat various human diseases including cancer, inflammation, and hereditary diseases such as LSDs^47–51^. While loading and delivery of biologically active lysosomal enzymes onto exosomes has been hypothesized, we are unaware of any demonstration of the feasibility or effectiveness of this approach. Lysosomal enzymes have a natural address (targeting lysosomes instead of exosomes). Moreover the activities of lysosomal enzymes require specific post-translational modifications, including glycosylation^44,45^. Consequently, the delivery of these enzymes to cell models of LSDs requires the development of a robust exosome-targeting scaffold that projects the enzyme with the correct geometry and allows for post-translational modifications during exosome biogenesis. To achieve this goal, we employed the single transmembrane protein, VSVG, which we have previously shown to be efficiently expressed in human cells. VSVG serves as a scaffold amenable to load proteins onto exosomes in a predefined manner by genetic fusion^42^. VSVG has a number of advantages for exosome targeting compared to other exosome-targeting scaffolds, including tetraspanins (CD9, CD63, and CD81), lipid anchoring peptide (C1C2 domain of lactacherin) and lysosome membrane protein (Lamp2B)^37,43,51,52^. First, VSVG integrates into exosomal membranes providing a stable anchoring mechanism for their fused-enzyme cargo. Thus, VSVG is not conceptually different from lysosome proteins and lipid anchors, neither of which is sufficiently stable nor can they be specifically targeted. Second, VSVG is a single transmembrane protein with a well-defined domain structure and membrane topology, composed of an ectodomain, a transmembrane helix, and a small luminal peptide^42^. This linear structure streamlines the design of geometrically-defined fusion proteins that may be directed to the outer surface or inside the lumen of the exosome. We note other scaffolds often lack this flexibility. For example, tetraspanins and C1C2 restrict their cargo loading to either the inside or the outside of the exosomal membrane^37,52^, while Lamp2B requires additional glycosylation to maintain the stability of its protein cargo^53^. Third, VSVG has a programmable ectodomain that can be easily swapped with cell-specific peptides or nanobodies, therefore permitting advanced engineering for additional benefits such as specific cell type targeting, or purification^42^.

Post-translational modification of GBA such as glycosylation is an important step in the production of a functional enzyme^44,45^. Glycosylation occurs co-translationally and is needed for the stability and lysosomal targeting, therefore endogenous activities in lysosomes^41,54–56^. However, our engineered GBA-fusion proteins still go through normal translation, the intrinsic enzyme activities may not be severely affected as evidenced by readily detected increased GBA activities in our transfected cells and EVs. It remains unknown if our GBA-fusion proteins are subjected to differentially co-translational glycosylations as compared to native GBA.

Another unique feature of this study is the genetic design used to produce modified exosomes. The exosomes present functional GBA enzymes in two different spatial conformations (either the outer or inner surface of exosome membrane), and the scaffold is capable of accommodating a reporter protein (GFP). GFP is especially useful in the observation of intracellular trafficking and uptake of purified exosomes in recipient cells. Interestingly, the two different placements of GBA enzyme lead to similar results. However, the two methods may have distinct advantages and potential limitations. The surface display approach may allow to load many copies of the enzyme to the exosome membrane surface, which may also increase the chance of enzymatic degradation during delivery. Placing enzymes within the lumen of the exosome could provide increased protection and potentially elicit fewer off-target interactions, as the therapeutic enzyme would not be able to interact with external molecules, including proteases. Nevertheless, we did not observe a significant difference between these two GBA configurations, suggesting both approaches are effective. Further confirmation of GBA-loaded EVs using either patients-derived GBA mutant fibroblast cells or *in vitro* GBA mutant cells as functional relevance is critical for further development. Additionally, studies using animal models are needed to determine which composition would result in the most effective therapeutic benefit.

While our exosome surface display is promising for treating Gaucher disease, there may be some *in vivo* limitations such as immunological responses to this biologics, which needs further studies using animal models.

## Conclusion

We have developed a genetic approach to produce enzyme-loaded EVs/exosomes for the targeted delivery of GBA enzyme to human cells. Through fusion with VSVG, the enzyme payloads are strongly enriched in EVs/exosomes, which are delivered to the endocytic compartments (early/late endosomes and lysosomes) of recipient cells. Our proof-of-concept work establishes the engineering methodology of EVs/exosomes for targeted delivery of bioactive lysosomal enzymes for a potential treatment strategy of Gaucher disease, especially for those with severe forms of the disease associated with neurological complications.

## Supplementary information

Supplementary Information
