# Supplementary material for: Targeted delivery of lysosomal enzymes to the endocytic compartment in human cells using engineered extracellular vesicles
Source: Sci Rep. 2019 Nov 21;9:17274. doi: 10.1038/s41598-019-53844-5 (PMC6872767; doi:10.1038/s41598-019-53844-5)
Supplement: Supplementary file 1 — Supplementary Information [file 41598_2019_53844_MOESM1_ESM.pdf]

Targeted delivery of lysosomal enzymes to the endocytic compartment in human cells using engineered extracellular vesicles

Mai Anh Do[1,#], Daniel Levy[1,#], Annie Brown[1], Gerard Marriott[2], Biao Lu[1,§]

[1]Deparment of Bioengineering, School of Engineering, Santa Clara University, 500 El Camino Real, Santa Clara, California 95053, USA. [2]Department of Bioengineering, University of California at Berkeley, Berkeley, CA94720, USA

[§]Corresponding author: blu2@scu.edu; Phone: 1-408-551-3510

Supplementary File 1

Supplementary Sequences

1. N-CD63-hTNFR1-EC-C-CD63-GFP coding sequences
2. N-CD63-hTNFR1-EC-C-CD63-GFP chimeric protein sequences

===

1. N-CD63-hTNFR1-EC-C-CD63-GFP coding sequences

ATGGCGGTGGAAGGAGGAATGAAATGTGTGAAGTTCTTGCTCTACGTCCTCCTGCTGGCCTTTTGCGCCTGTGCAG
TGGGACTGATTGCCGTGGGTGTCGGGGCACAGCTTGTCCTGAGTCAGACCATAATCCAGGGGGCTACCCCTGGCT
CTCTGTTGCCAGTGGTCATCATCGCAGTGGGTGTCTTCCTCTTCCTGGTGGCTTTTGTGGGCTGCTGCGGGGCCTG
CAAGGAGAACTATTGTCTTATGATCACGTTTGCCATCTTTCTGTCTCTTATCATGTTGGTGGAGGTGGCCGCAGCCA
TTGCTGGCTATGTGTTTAGAGATAAGGTGATGTCAGAGTTTAATAACAACTTCCGGCAGCAGATGGAGAATTACCC
GAAAAACAACCACACTGCTTTCGAATCTGGCATGGGCCTCTCCACCGTGCCTGACCTGCTGCTGCCACTGGTGCTC
CTGGAGCTGTTGGTGGGAATATACCCCTCAGGGGTTATTGGACTGGTCCCTCACCTAGGGGACAGGGAGAAGAG
AGATAGTGTGTGTCCCCAAGGAAAATATATCCACCCTCAAAATAATTCGATTTGCTGTACCAAGTGCCACAAAGGA
ACCTACTTGTACAATGACTGTCCAGGCCCGGGGCAGGATACGGACTGCAGGGAGTGTGAGAGCGGCTCCTTCACC
GCTTCAGAAAACCACCTCAGACACTGCCTCAGCTGCTCCAAATGCCGAAAGGAAATGGGTCAGGTGGAGATCTCT
TCTTGCACAGTGGACCGGGACACCGTGTGTGGCTGCAGGAAGAACCAGTACCGGCATTATTGGAGTGAAAACCTT
TTCCAGTGCTTCAATTGCAGCCTCTGCCTCAATGGGACCGTGCACCTCTCCTGCCAGGAGAAACAGAACACCGTGT
GCACCTGCCATGCAGGTTTCTTTCTAAGAGAAAACGAGTGTGTCTCCTGTAGTAACTGTAAGAAAAGCCTGGAGTG
CACGAAGTTGTGCCTACCCCAGATTGAGAATGTTAAGGGCACTGAGGACTCAGGCACCACAGGGCTCGATTTAAA
TTCGATCCTGGACAGGATGCAGGCAGATTTTAAGTGCTGTGGGGCTGCTAACTACACAGATTGGGAGAAAATCCC
TTCCATGTCGAAGAACCGAGTCCCCGACTCCTGCTGCATTAATGTTACTGTGGGCTGTGGGATTAATTTCAACGAG
AAGGCGATCCATAAGGAGGGCTGTGTGGAGAAGATTGGGGGCTGGCTGAGGAAAAATGTGCTGGTGGTAGCTG
CAGCAGCCCTTGGAATTGCTTTTGTCGAGGTTTTGGGAATTGTCTTTGCCTGCTGCCTCGTGAAGAGTATCAGAAG
TGGCTACGAGGTGATGatggagagcgacgagagcggcctgcccgccatggagatcgagtgccgcatcaccggcaccctgaacggcgtggag
ttcgagctggtgggcggcggagagggcacccccaagcagggccgcatgaccaacaagatgaagagcaccaaaggcgccctgaccttcagcccctac
ctgctgagccacgtgatgggctacggcttctaccacttcggcacctaccccagcggctacgagaaccccttcctgcacgccatcaacaacggcggcta
caccaacacccgcatcgagaagtacgaggacggcggcgtgctgcacgtgagcttcagctaccgctacgaggccggccgcgtgatcggcgacttcaag
gtggtgggcaccggcttccccgaggacagcgtgatcttcaccgacaagatcatccgcagcaacgccaccgtggagcacctgcaccccatgggcgata
acgtgctggtgggcagcttcgcccgcaccttcagcctgcgcgacggcggctactacagcttcgtggtggacagccacatgcacttcaagagcgccatc
caccccagcatcctgcagaacgggggccccatgttcgccttccgccgcgtggaggagctgcacagcaacaccgagctgggcatcgtggagtaccagc
acgccttcaagacccccatcgccttcgccagatcccgcgctcagtcgtccaattctgccgtggacggcaccgccggacccggctccaccggatctcgcC
ATCATCATCATCATCATTAA

Note: N-CD63: N-terminus of CD63 coding sequences; C-CD63: C-terminus of CD63 coding sequences; hTNFR1-EC: human TNFα receptor 1 extracellular domain coding sequences; GFP: green fluorescent protein coding sequences

2. N-CD63-hTNFR1-ED-C-CD63-GFP chimeric protein sequences

MAVEGGMKCVKFLLYVLLLAFCACAVGLIAVGVGAQLVLSQTIIQGATPGSLLPVVIIAVGVFLFLVAFVGCCGACKENY
CLMITFAIFLSLIMLVEVAAAIAGYVFRDKVMSEFNNNFRQQMENYPKNNHTAFESGMGLSTVPDLLLPLVLLELLVGIY
PSGVIGLVPHLGDREKRDSVCPQGKYIHPQNNSICCTKCHKGTYLYNDCPGPGQDTDCRECESGSFTASENHLRHCLSC
SKCRKEMGQVEISSCTVDRDTVCGCRKNQYRHYWSENLFQCFNCSLCLNGTVHLSCQEKQNTVCTCHAGFFLRENECV
SCSNCKKSLECTKLCLPQIENVKGTEDSGTTGLDLNSILDRMQADFKCCGAANYTDWEKIPSMSKNRVPDSCCINVTVG
CGINFNEKAIHKEGCVEKIGGWLRKNVLVVAAAALGIAFVEVLGIVFACCLVKSIRSGYEVMMESDESGLPAMEIECRIT
GTLNGVEFELVGGGEGTPKQGRMTNKMKSTKGALTFSPYLLSHVMGYGFYHFGTYPSGYENPFLHAINNGGYTNTRIE
KYEDGGVLHVSFSYRYEAGRVIGDFKVVGTGFPEDSVIFTDKIIRSNATVEHLHPMGDNVLVGSFARTFSLRDGGYYSFV
VDSHMHFKSAIHPSILQNGGPMFAFRRVEELHSNTELGIVEYQHAFKTPIAFARSRAQSSNSAVDGTAGPGSTGSRHH
HHHH

Note: N-CD63: N-terminus of CD63; C-CD63: C-terminus of CD63; hTNFR1-EC: human TNFα receptor 1-extracellular domain ; GFP: green fluorescent protein

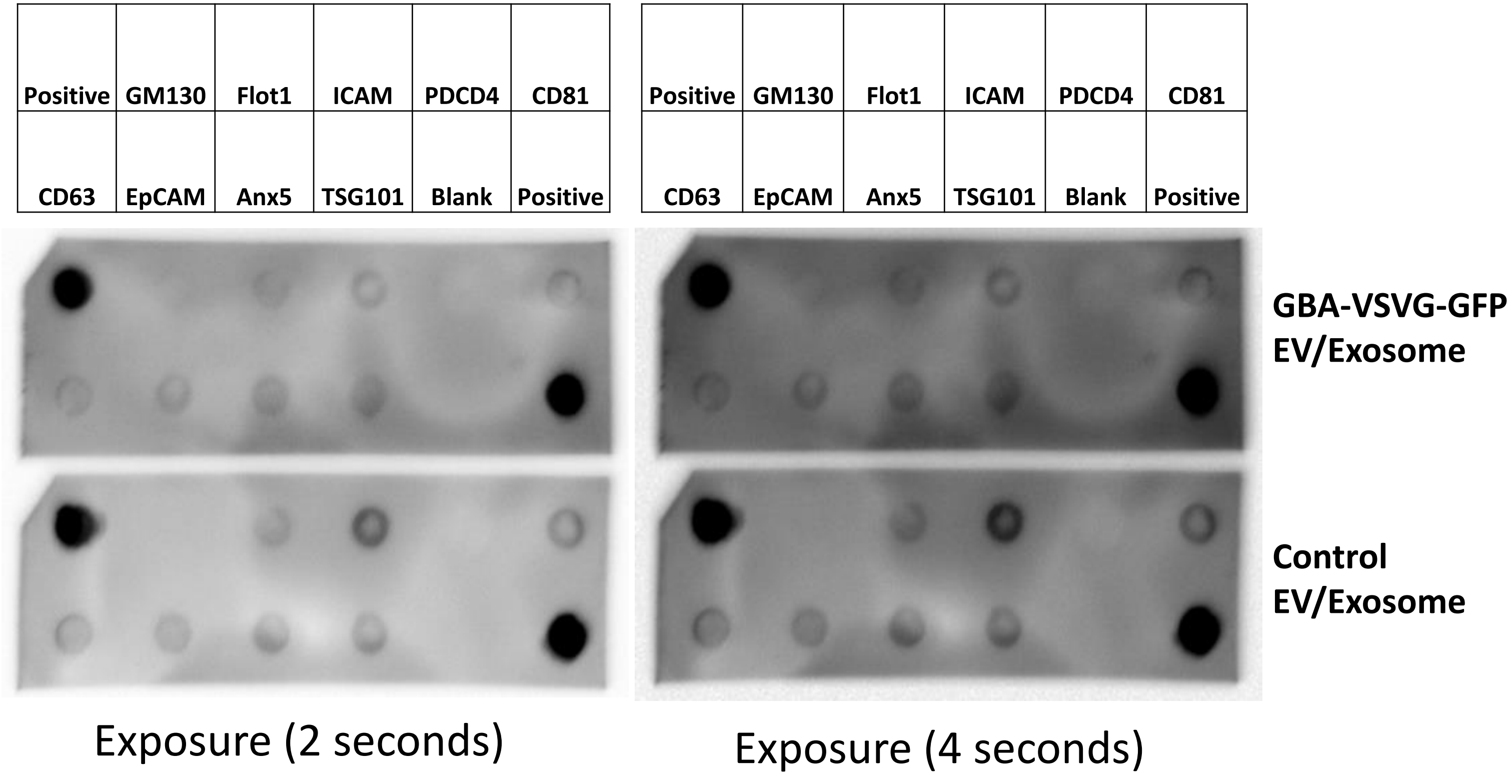

Figure S1. Characterization of engineered extracellular vesicle (EV) by a dot-blot array analysis is shown in two exposure conditions. Dot-blot array analysis of 9 exosomal markers shows similar expression pattern of engineered exosomes from cells transfected with GBA-VSVG-GFP (b; upper panels) vs. non-modified control (b; lower panels) using ChemiDoc XRS1 could charged –coupled device (BioRAD, USA).

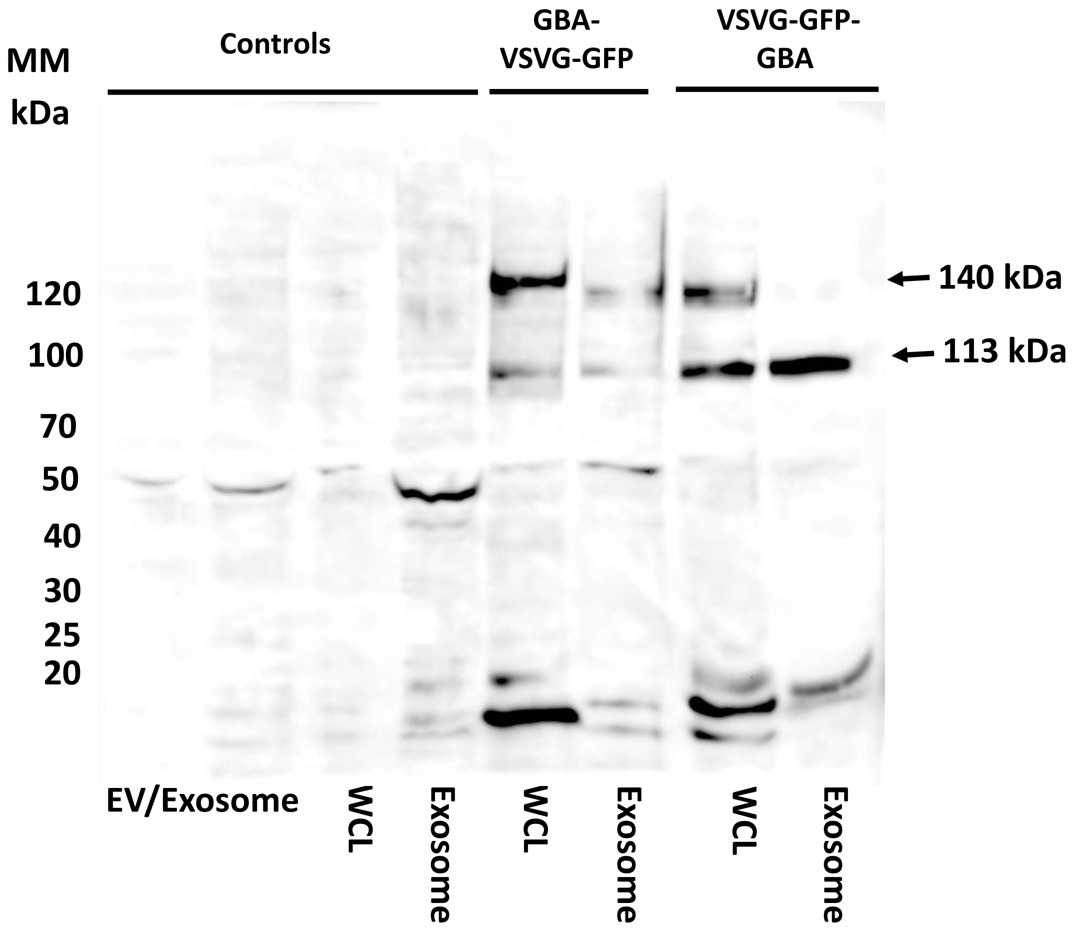

Figure S2. Characterization of engineered EV/exosome by Western blot analysis. The full-blot including multiple control EV/exosome preparations is shown. The membrane was visualized with Pierce ECL Western Blotting Substrate on ImageQuant LAS 500 imager (GE Healthcare Life Sciences; Issaquah, WA).

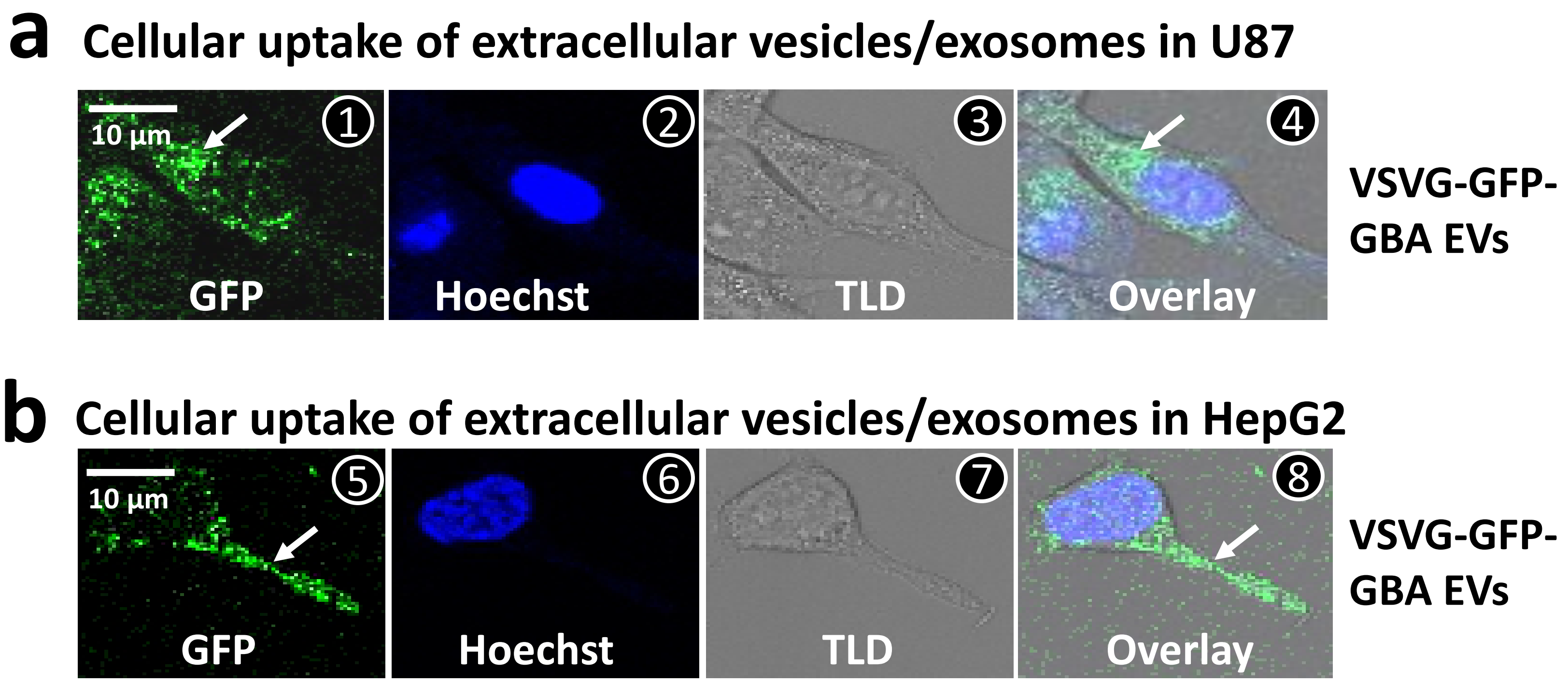

Figure S3. Delivery of GBA extracellular vesicles/exosomes to U87 and HepG2 cells. Parental U87 cells were treated with VSVG-GFP-GBA loaded extracellular vesicles/exosomes in a glass bottom culture dish. 48 hours post-treatment, fluorescent, Hoechst stained, and PMT-Trans (TLD) images were taken and overlaid (**a1-4, upper panels**). The same procedure was performed in HepG2 cells such that the uptake of VSVG-GFP-GBA loaded extracellular vesicles/exosomes was observed via confocal microscopy (**b5-8, lower panels**). EV, extracellular vesicle.
